# A pilot study investigating the influence of oxytocin on attentional bias to food images in women with bulimia nervosa or binge eating disorder

**DOI:** 10.1111/jne.12843

**Published:** 2020-03-23

**Authors:** Monica Leslie, Jenni Leppanen, Yannis Paloyelis, Janet Treasure

**Affiliations:** ^1^ Institute of Psychiatry, Psychology and Neuroscience (IoPPN) King’s College London (KCL) London UK

**Keywords:** attentional bias, binge eating disorder, bulimia nervosa, eating disorders, oxytocin

## Abstract

**Background:**

Previous research has found that exogenous oxytocin administration has the potential to modulate attentional biases in women with anorexia nervosa. Recent work has indicated that attentional biases to food may reinforce the recurrent binge eating behaviour characterising bulimia nervosa and binge eating disorder. To date, however, no study has yet investigated the effect of oxytocin on attentional biases to palatable food in women with bulimia nervosa and binge eating disorder.

**Methods:**

The present study employed a single‐session cross‐over design to test the hypothesis that a divided dose of 64 IU of intranasal oxytocin, administered as one intranasal dose of 40 IU of oxytocin followed by a top‐up of 24 IU of oxytocin 80 minutes later, vs placebo administration administered in the same dosing schedule would reduce attentional biases towards food images in a dot probe task. We hypothesised that oxytocin administration would reduce vigilance towards food to a greater degree in women with bulimia nervosa or binge eating disorder vs healthy comparison women. Twenty‐five women with bulimia nervosa or binge eating disorder and 27 comparison women without history of an eating disorder were recruited to take part in the study.

**Results:**

In contrast to our hypothesis, there was no main effect of diagnosis on attentional bias to food (fixed effect = 5.70, *P* = 0.363), nor a significant interaction between diagnosis and drug condition (fixed effect =−14.80, *P* = 0.645). There was a main effect of drug condition, such that oxytocin increased vigilance towards food vs neutral images in the dot probe task (fixed effect = 10.42, *P* = 0.044). A correlation analysis revealed that this effect was moderated by attentional bias in the placebo condition, such that greater avoidance of food stimuli in the placebo condition was associated with a greater increase in vigilance induced by oxytocin.

**Conclusions:**

The findings of the present study add to a mixed body of literature investigating the therapeutic effects of oxytocin in women. Future research would benefit from dose‐response studies investigating the optimal dose of oxytocin for modulating the attentional processing of palatable food in populations with eating disorders.

## 1 INTRODUCTION

Bulimia nervosa (BN) and binge eating disorder (BED) are Diagnostic and Statistical Manual of Mental Disorders (DSM)‐5 eating disorders characterised by recurrent, loss‐of‐control binge eating behaviour over a period of at least 3 months.^1^ Currently, the average remission rate for people with BN and BED remains low and treatment presents a significant challenge.^2^ The development of new treatment approaches is therefore warranted to address this unmet need.

A meta‐analysis has found that people with BN exhibit greater vigilance towards food words in Stroop tasks compared to participants without history of an eating disorder.^3^ More recently, evidence has supported the hypothesis that hypervigilance towards food cues is associated with core eating disorder behaviours and psychopathology in BN and BED. For example, Albery et al^4^ used a Stroop task to measure attentional bias to both food‐ and body‐related words amongst women with BN. They found that the degree of hypervigilance towards food‐related words was associated with greater frequency of binge‐purge behaviour. Additionally, Svaldi et al^5^ used a visual priming task to investigate attentional bias to food images vs neutral images among obese participants with and without BED. Although this priming effect ultimately did not differ between the BED and non‐BED groups of overweight participants, they did find that the degree of priming was positively correlated with overall eating disorder psychopathology, as measured using the Eating Disorder Examination‐ Questionnaire (EDE‐Q).^6^ In a spatial cueing task, Schmitz et al^7^ also found the same positive correlation between vigilance to food images and EDE‐Q scores in a sample of participants with BED.

It has been proposed that attentional bias toward palatable food cues is a cognitive maintenance factor for BN and BED because these preconscious biases enhance the salience of external cues for binge eating.^8^ Evidence suggests that the link between attention to food cues and subsequent binge eating behaviour may be especially strong in these disorders as a result of an automatic stimulus‐response association that becomes increasingly reinforced with continued binge eating behaviour.^9^, ^10^ Previous evidence has indicated that attentional biases to food are especially strong following actual food consumption in populations with obesity, compared to healthy control participants.^11^ However, the effect of food consumption on behavioural measures of attentional bias to food has not been investigated in people with BN and BED.^12^ It is therefore of interest to determine whether hypervigilance to palatable food occurs prior to food consumption, thus supporting trait levels of heightened incentive salience in BN and BED,^13^ or whether this hypervigilance only or also occurs following food consumption, thus potentially highlighting that the onset of food consumption triggers subsequent increases in incentive salience.

Oxytocin is a hormone and neuropeptide commonly known for its roles in modulating social perception and behaviour.^14^ However, in recent years, oxytocin has gained increasing attention for its effects on eating in both healthy and clinical populations.^15^ Recent research has suggested that the hormone oxytocin may affect the upstream cognitive and emotional processes that contribute to the maintenance of disordered eating behaviour in anorexia nervosa. For example, chronic administration of oxytocin has been found to reduce eating concern^16^ and correct attentional avoidance of food images in women with anorexia nervosa.^17^ This finding potentially carries clinical significance given evidence that attentional avoidance of food stimuli is correlated with disorder severity in anorexia nervosa,^18^ although future research is required before causal effects of attentional bias can be determined. Previous research has found that exogenous oxytocin administration also reduces vigilance to angry faces, an anxiety‐provoking social stimulus, in women with BN.^19^ To our knowledge, however, no study has yet investigated the effects of oxytocin on attentional bias to food images in BN and BED.

It is not entirely clear by what mechanism oxytocin alters attentional bias specifically to food images in anorexia nervosa, although the strength of the effect of oxytocin on attentional bias is related to the anxiolytic effects of oxytocin with a medium effect size.^17^ It may be the case that anxiety primarily drives baseline attentional biases away from food (avoidance) in anorexia nervosa, and the oxytocin‐induced reduction in anxiety has the downstream effect of normalising this bias. A current lack of evidence does not allow for the establishment of firm conclusions regarding the mechanism of the effect of oxytocin on attentional bias; however, it is reasonable to suspect that oxytocin may exert a similar effect in normalising baseline attentional biases towards food (vigilance) in BN and BED if oxytocin modulates a common anxiety‐based mechanism accounting for baseline attentional biases in each disorder, albeit in opposite directions.

Previous evidence that intranasal oxytocin suppresses hedonic eating in overweight men has provided tentative evidence that oxytocin may also suppress the reward salience of palatable food,^20^ which has previously been found to affect preconscious attentional bias.^21^ Furthermore, previous neuroimaging work has found that people recovered from BN exhibit similar blood‐oxygenated‐level‐dependent (BOLD) responses within the left putamen, a neural region strongly associated with the processing of reward, in response to taste stimuli received when either hungry or sated.^22^ This is in contrast to healthy control participants, who exhibit a down‐regulated BOLD response in the left putamen following food consumption.^22^ One potential hypothesis stemming from this pattern of effects is that the incentive salience of palatable food, and attentional bias to palatable food, will decrease following food consumption to a greater degree amongst healthy control participants, compared to participants with BN or BED. Furthermore, previous evidence finding that increased activation of oxytocinergic neurones within the paraventricular nucleus of the hypothalamus precedes meal termination suggests that the effect of oxytocin in deterring food‐seeking, and thus attentional orienting towards food stimuli, may be stronger following food consumption given the role of oxytocin in meal termination.^23^

The current pilot used a double‐blind, placebo‐controlled cross‐over design to test the effect of a divided dose of 64 IU of intranasal oxytocin on attentional bias to food images among women with BN or BED and comparison women without history of an eating disorder. Our hypotheses were: (i) women with BN or BED would demonstrate greater vigilance towards food images than women without history of an eating disorder; (ii) oxytocin administration would reduce vigilance towards food images in both groups of women, based on previous work suggesting that oxytocin reduces the incentive salience of palatable food in healthy participants^24^; (iii) oxytocin would reduce vigilance towards food images to a greater degree in women with BN or BED vs healthy comparison women as a result of potential flooring effects in the healthy comparison group; and (iv) there would be an interaction between time point and participant group, such that the difference in attentional bias to food in the BN/BED vs the healthy control group would be even greater following food consumption.

## 2 MATERIALS AND METHODS

### 2.1 Participants

Fifty‐two women participated in the present study. Given that recurrent binge eating behaviour was the primary trait of interest in the present study, and that populations with BN and BED are both characterised by recurrent loss‐of‐control binge eating behaviour, we therefore recruited a heterogeneous sample of women who met criteria for either disorder. Twenty women met DSM‐5 diagnostic criteria for BN, five women met DSM‐5 diagnostic criteria for BED and 27 women had no prior history of an eating disorder at the time of the study. An a priori power analysis conducted in gpower (http://www.gpower.hhu.de) indicated that a sample size of 20 participants per group would be sufficient to detect a mean difference between groups with a medium effect size. Therefore, the sample size included in the present study had more than the minimum number of participants indicated by the power analysis. The study was advertised on the website for a major eating disorder charity in the United Kingdom (Beat) (https://www.beateatingdisorders.org.uk), via e‐mail circulars at King's College London and on flyers displayed on community bulletin boards. The London‐Camberwell St Giles NHS Research Committee granted ethical approval for the present study (reference: 14/LO/2115). All participants provided their written informed consent in accordance with the Declaration of Helsinki. Full inclusion and exclusion criteria for the study are presented in the Supporting information (Appendix S1). Eligibility for the present study was established via a phone screening, which included the Structured Clinical Interview for DSM‐5 (Research Version).^25^

Seven participants who met diagnostic criteria for BN or BED self‐reported at least one comorbid psychiatric disorder. Specifically, five women had comorbid depression, four women had borderline personality disorder, four women had comorbid generalised anxiety disorder, one woman had obsessive‐compulsive disorder, one woman had social anxiety and one woman had an autism spectrum disorder. At the time of the study, seven women were taking an antidepressant, one woman was taking an antipsychotic drug and one woman was taking a mood stabiliser. Twenty‐two of the fifty‐two participants were taking hormonal contraception at the time of the study. Fifteen women completed the study when they were in the follicular phase of the oestrous cycle and thirteen women completed the study in the luteal phase of the menstrual cycle. There was no significant difference between the BN/BED and healthy comparison participant groups in the distribution of women in the follicular phase, luteal phase, or on hormonal contraception (χ^2^ = 4.61, *df* = 2, *P* = 0.100). Menstrual phase data were missing for two women. Descriptive statistics for the age, body mass index and education level of the participant sample are presented in Table 1.

**Table 1:** Descriptive demographic data

	Healthy control (n = 27)		BN/BED (n = 25)	
	Median	IQR	Median	IQR
Age	23.50	5.50	23.50	9.75
BMI	22.04	1.76	23.09	3.87
RQF education level	6	4	4.5	3

Abbreviations: BED, binge eating disorder; BMI, body mass index; BN, bulimia nervosa; IQR, interquartile range; RQF, Regulated Qualifications Framework.

### 2.2 Study design

The study used a double‐blind placebo‐controlled cross‐over design. Each participant came to the laboratory for three study visits: one orientation visit and two experimental sessions. During the orientation visit, each participant had the opportunity to discuss any queries with the researcher in person before signing informed consent and practising self‐administration of a placebo nasal spray. The height and weight of each participant were also measured during the orientation visit. At the conclusion of the orientation visit, each participant was provided with a link to an online survey, in which participants provided basic demographic data prior to the second study visit.

Each participant subsequently came to the laboratory for two experimental study visits. Participants received a divided dose of 64 IU of intranasal oxytocin (Syntocinon, 40 IU mL^‐1^; Novartis, Basel, Switzerland) during one experimental study visit, and an equal volume of a placebo nasal spray (containing the same excipients as the oxytocin nasal spray but without the active ingredient of oxytocin) during the opposite visit. The order in which each participant received each nasal spray was pseudo‐randomised, such that an equal number of participants received oxytocin on the first vs the second experimental study visit. Both the participant and the researcher were blind to the order in which each participant was allocated the oxytocin vs placebo nasal spray. These experimental study visits were held 2 days apart to ensure that each participant would complete both experimental study visits when they were in the same phase of the menstrual cycle. Perfusion data analysing the central effects of oxytocin suggest that carry‐over effects from oxytocin administration on the first experimental visit to the second experimental visit are highly unlikely.^26^

Participants were requested to abstain from consuming alcohol or caffeine from 8.00 pm on the evening prior to each experimental study visit. Participants were also asked to eat 2.5 hours prior to the beginning of each experimental study visit, and nothing else between this time and the beginning of the experimental study visit.

Participants arrived for each study visit at 5.00 pm. At 5.50 pm, each participant self‐administered the first dose of the allocated nasal spray for that day: either 40 IU of oxytocin or an equal volume of the placebo nasal spray. Each participant then underwent a functional magnetic resonance imaging (fMRI) scan, during which they viewed images of water and chocolate milk and received 0.5‐mL doses of water and chocolate milk via a tube. The results of this fMRI analysis are not reported in the present study. We chose to administer a 40‐IU dose of oxytocin prior to the fMRI scan to replicate the previous protocol of Kim et al^27^, which found an effect of oxytocin on eating behaviour in women with BN at this dose. We additionally chose this dose because previous work has found that 40 IU of intranasal oxytocin effectively engages target regions associated with the neural processing of taste reward.^26^

**Figure 1:**
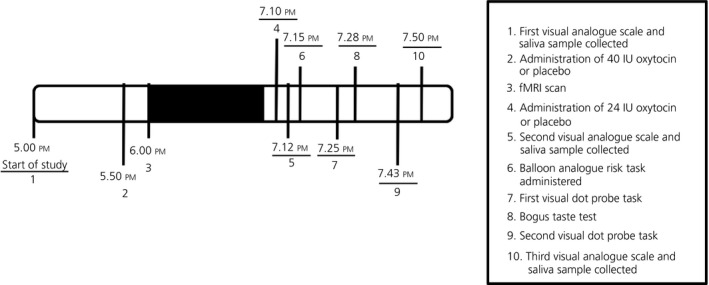
Timeline of study events. fMRI, functional magnetic resonance imaging

The functional MRI scan ended at 7.00 pm, 65 minutes after the completion of initial drug administration. Previous findings in humans have suggested that peak central effects of 40 IU intranasal oxytocin occur 39‐51 minutes following administration,^26^ thus suggesting the need for an additional dose after this time. Therefore, at 7.10 pm, participants self‐administered a second dose of the allocated nasal spray for that day: either 24 IU of oxytocin or an equal volume of the placebo nasal spray. At 7.25 pm, each participant then completed a visual dot probe task, which is described in further detail below. Immediately following the completion of this task, each participant completed a “bogus” taste test, which is also described below. The taste test continued for a standard 15‐minute duration. Following the taste test, each participant repeated the visual dot probe task. Subsequently, each participant reported whether they believed they had been allocated the oxytocin or placebo for that study visit. A timeline of the primary events which took place during each experimental visit is presented in Figure 1.

### 2.3 Visual dot probe task

The visual dot probe task is a common test of attentional bias to disorder‐relevant images, which has been used to measure attentional biases across a range of psychiatric disorders.^28^ The visual dot probe task used in the present study was adapted from the study reported by Cardi et al^29^ and presented using e‐prime (Psychology Software Tools, Sharpsburg, PA, USA). Each task run consisted of 96 trials. In each trial, the participant was first presented with two images side by side, with 115 mm between the centres of each image. Each image belonged to one of two category types: food images (32 different images) or neutral images (48 different images). Food images depicted close‐up photographs of palatable foods on a plate. Food was depicted in a “ready‐to‐eat” form without any packaging.

Participants were presented with a total of 32 different food‐neutral image pairs, and 16 different neutral‐neutral image pairs. Each image pair was presented twice (once with the food picture on the left, and once with the food picture on the right; the order of image pairings was randomly determined for each participant). All food images were matched for size and caloric content. Neutral images depicted furniture. Each image had a resolution of 72 dpi, measured 45 × 70 mm on the computer screen, and was matched for colour saturation. Each picture pair was presented for 500 ms.

Immediately following the presentation of each image pair, one of the images was replaced by a visual probe. Visual probes consisted of either a pair of horizontally or vertically oriented dots. Participants were instructed to press the letter ‘z’ as soon as they saw a pair of horizontally oriented dots, or the letter ‘q’ as soon as they saw a pair of vertically oriented dots. Stickers depicting the corresponding dot orientation were attached to the letters ‘q’ and ‘z’ on the keyboard to prevent confusion for participants. The inter‐trial interval was 500 ms. Reaction time and accuracy were recorded for each trial.

### 2.4 Bogus taste test

The bogus taste test is a validated measure of eating behaviour used to test the effect of experimental factors on the consumption of palatable food.^30^ In the present study, each participant was presented with three types of food, each of which was contained in a standardised white ceramic bowl. One bowl contained 250 g of grapes, one bowl contained 50 g of Walker's ready‐salted crisps (Walkers Snacks, Leicester, UK), and the final bowl contained 220 g Cadbury Bitsa Wispa™ chocolate (Cadbury UK, Birmingham, UK). The bowls were presented in a standard order, with the bowl of grapes being the leftmost bowl, followed by the bowl of crisps, and the bowl of chocolate being the rightmost bowl. Participants were asked to rate each type of food for tastiness, sweetness, saltiness, richness, and pleasantness on a visual analogue scale anchored from 0 to 10. As the researcher was leaving the room after explaining the instructions for the bogus taste test, the researcher told each participant as an “afterthought” that the participant was welcome to eat as much as they would like, as the remaining food would be thrown away. Participants were then left alone in a room with the three bowls of food for 15 minutes to complete the task.

Following the completion of the bogus taste test, the three bowls of food were brought to a separate room, out of sight of the participant, where a research assistant weighed the remaining food in each bowl and subtracted this amount from the initial weight of each type of food. The effect of oxytocin on the quantity of food eaten is reported elsewhere.^31^

### 2.5 Statistical analysis

Attentional bias scores for each task run were calculated for the dot probe task by subtracting each participant's mean reaction time to probes that were preceded by a food image from those that were preceded by a neutral image. Therefore, positive attentional bias scores indicate vigilance to food images, and negative attentional bias scores indicate avoidance of food images. Trials that presented matching image pairs (eg, two neutral images) and trials in which the participant responded incorrectly were excluded. All task runs for all participants met the minimum requirement of an 80% correct response rate.

We tested our hypotheses using a 2 × 2 × 2 linear mixed effects model in the lmerTest package for r.^32^ We included experiment time point (before or after the taste test), eating disorder status (healthy controls or BN/BED), drug condition (placebo or oxytocin) and all associated interactions as fixed effects. The intercept for each participant was entered as a random effect. A preliminary linear mixed effects analysis was conducted to determine whether hormonal state (follicular phase, luteal phase or current hormonal contraception) moderated the effect of oxytocin on attentional bias. The results of this linear mixed effects analysis are reported in the Supporting information (Table S1). The interaction effect between oxytocin and hormonal state was not significant, and hormonal state was therefore not included as a covariate in the final mixed effects model. We also conducted an additional linear mixed effects analysis to determine whether there was a significant effect of learning on attentional bias by comparing attentional bias between the participants’ first and second visits. There was no significant simple main effect of visit number on attentional bias (estimate = −7.16, *SE* = 5.19, *df* = 141.98, *t* = −1.38, *P* = 0.170), nor was there a significant interaction between visit number and drug condition or visit number and eating disorder status on attentional bias. The full results of the interaction analysis are presented in the Supporting information (Table S2).

## 3 RESULTS

The attentional bias scores were first screened for outliers and violations of the assumption of normality. Four outliers (|*Z|*> 3.0) were found in the attentional bias variable and excluded from subsequent analyses. Three of these outlier data points were in the BN/BED participant group and one was in the healthy control participant group. Descriptive statistics associated with the attentional bias scores are presented in Table 2, and visual depictions of the data are presented in the Supporting information (Figures S1 and S2).

**Table 2:** Results of the linear mixed effects analysis testing the main effects of eating disorder status, oxytocin, and food presentation (experiment time point) on attentional bias to food images

		HC (n = 27) Mean ± SD	BN/BED (n = 25) Mean ± SD	Fixed effects
Before taste test	Oxytocin	10.07 ± 37.200	10.54 ± 39.720	ED status: *Z* = 5.70, *SE* = 6.206, *df* = 46.00, *P* = 0.363 Drug condition: *Z* = 10.42*, *SE* = 5.136, *df* = 142.20, *P* = 0.044 Experiment time point: *Z* = 1.10, *SE* = 5.14, *df* = 143.76, *P* = 0.831
	Placebo	‐0.53 ± 38.897	0.40 ± 49.889	
After taste test	Oxytocin	2.74 ± 31.922	19.93 ± 42.467	
	Placebo	‐0.26 ± 26.770	2.29 ± 36.677	

				Random effects	Variance
				Individual participant	151
				Residuals	1284

Abbreviations: BED, binge eating disorder; BN, bulimia nervosa; ED, eating disorder; HC, healthy control.

* *P* < .05

The linear mixed model found that none of the interaction effects reached significance in the full factorial model. The fixed effects associated with the full factorial linear mixed effects analysis are presented in Table 3. We did not identify any cases with excessive influence on the model (all Cook's distances <1.0).

**Table 3:** Results of the linear mixed effects analysis testing a full factorial model with fixed effects for eating disorder status, oxytocin, and food presentation (experiment time point) on attentional bias to food images

	Estimate	*SE*	*df*	*t*	*P*
Fixed effects					
Intercept	−0.45	16.170	163.81	−0.03	0.978
ED status	−1.75	22.868	163.86	−0.08	0.939
Drug condition	17.85	22.536	141.17	0.79	.0430
Experiment time point	−0.08	10.169	142.17	−0.01	0.994
ED Status × drug condition	−14.80	32.083	141.71	−0.46	0.645
ED Status × experiment time point	2.68	14.382	142.30	0.19	0.853
Drug condition × experiment time point	−7.25	14.301	141.55	−0.51	0.613
ED status × drug condition × experiment time point	14.62	20.390	141.91	0.72	0.475
Random effects	Variance				
Individual participant	159.8				
Residuals	1263.9				

Abbreviation: ED, eating disorder.

Because there were no significant interaction effects in the full factorial model, we therefore conducted a follow‐up linear mixed effects analysis including only main effects. The main effects analysis revealed that neither eating disorder status, nor experiment time point significantly affected attentional bias. However, there was a significant main effect of drug condition, such that oxytocin administration vs placebo administration was associated with significantly greater vigilance towards food images. The fixed effects associated with the linear mixed effects analysis are presented in Table 2. Again, we did not identify any cases with excessive influence on the model (all Cook's distances <1.0).

Visual inspection of the data revealed a numerically greater difference in attentional bias to food images between the oxytocin and placebo conditions among participants with BED, as opposed to healthy control or BN participant groups. We therefore conducted a sensitivity analysis excluding BED participants. Given that this exclusion would substantially reduce power in light of an already low sample size, we imputed the mean for each drug condition and experiment time point among the BN participant group for an additional five simulated BN participants. After excluding participants with BED, the effect of drug condition was no longer significant (*P* = 0.084) although there was a trend towards an effect in the same direction. The full results of this sensitivity analysis are reported in the Supporting information (Table S3).

Finally, given that we hypothesised that we would observe group differences in the effect of oxytocin on attentional biases driven by a flooring effect in the healthy control group, even in the absence of these group differences, we were interested in exploring whether baseline attentional bias to food stimuli in the placebo condition was associated with the degree of change induced by oxytocin administration. The attentional bias variable in the placebo condition and the attentional bias difference variable were approximately normally distributed at both Time Point 1 and Time Point 2 (skew < 2.0 and kurtosis < 2.0 for all variables). We therefore considered it appropriate to conduct a Pearson's correlation test. This correlation analysis revealed that greater avoidance of food images in the placebo condition was associated with a greater increase in attentional bias induced by oxytocin at both Time Point 1 (*r* = 0.70, N = 49, *P* < .001) and Time Point 2 (*r* = 0.65, N = 47, *P* < .001). The scatter plots associated with the correlation analyses for Time Point 1 and Time Point 2 are presented in the Supporting information (Figure S3 and Figure S4). We also conducted an exploratory linear mixed effects analysis investigating whether the number of calories consumed in the taste test impacted attentional bias or the effect of oxytocin on attentional bias. This linear mixed effects analysis did not reveal any significant main or interaction effects with experiment time point or drug condition. The full results of this analysis are reported in the Supporting information (Table S4).

## 4 DISCUSSION

The present study aimed to test the effect of a divided dose of 64 IU of intranasal oxytocin on attentional bias to food images in women with and without BN or BED. We hypothesised that women with BN or BED would demonstrate greater vigilance towards food images and that oxytocin administration would reduce vigilance towards food images in both participant groups, although with a stronger effect in the BN/BED vs the healthy comparison group.

Our first hypothesis was not supported by the results because there was no main effect of eating disorder status on attentional bias to food images. Oxytocin was found to have a significant effect on attentional bias; however, this was in the opposite direction to our hypothesis. That is, oxytocin was found to increase vigilance to food images; however, this effect was moderated by baseline attentional biases, such that greater avoidance of food stimuli in the placebo condition was associated with greater increases in vigilance induced by oxytocin. The main effect of oxytocin, associated with an increase in vigilance to food images, was primarily driven by the five participants with BED because the significance of this effect did not survive after excluding participants with BED. Our third hypothesis was also not supported by the results because there was no significant interaction between oxytocin treatment and eating disorder status on attentional bias to palatable food. Finally, we did not find evidence of an interaction between participant group and time point (before or after the taste test) on attentional bias to food images.

The lack of difference in baseline attentional bias to food stimuli between women with vs without BN or BED is in contrast to previous studies using Stroop tasks.^3^ This contrast in findings may partly be a result of differences in the food‐related stimuli presented: food images being used in the present study vs food words in the Stroop task. However, previous evidence has suggested that food images are rather associated with a greater difference in attentional bias to food amongst participants with eating disorders and healthy controls compared to word stimuli.^33^ The null effect of eating disorder status in the present study may therefore rather be a result of the stage of attention processing targeted in the present study. The target in this task was presented 500 ms after each image pair, although the nature of the Stroop task necessarily requires that the target be presented simultaneously with the food‐related word. This is potentially relevant given previous eye‐tracking evidence finding no difference in attention to food images among women with anorexia nervosa at early stages of attentional processing but avoidance of food images at later stages of attentional processing.^18^ If similar variation in attentional biases also exists in populations with BN and BED, it is possible that the present study failed to detect differences in attentional bias before or after the target appeared.

The finding that oxytocin induced greater, rather than less, vigilance towards food stimuli in the present study was surprising, and contrasted with our hypothesis that oxytocin would reduce vigilance to food stimuli across all study participants. However, it should be noted that the increased vigilance observed in the present study was not robust to the exclusion of the five participants with BED, even after imputing the mean for five simulated participants with BN to make up for the loss of power. Although the sample of participants with BED was not sufficiently large to warrant a moderation analysis comparing the influence of oxytocin on participants with BN vs BED, these preliminary results demonstrating numerical divergence in the effect of oxytocin on attentional bias to palatable food among each disorder suggest that attentional biases to palatable food should be investigated separately among samples with BN and BED in future studies. On the whole, given that baseline attentional biases in women with anorexia nervosa have previously been found to be away from food images,^17^ rather than towards food images, the evidence to date suggests that, on average, oxytocin increases vigilance to food images in women, with correlation analyses suggesting that this effect is greater in participants with baseline attentional avoidance of food stimuli.

The mechanism by which oxytocin increases attentional bias to food images in women is yet unclear, although it may involve interactions with dopaminergic signalling systems in mesolimbic brain regions.^34^ Specifically, binding to oxytocin receptors in the ventral tegmental area and nucleus accumbens, two regions strongly related to reward processing, may initiate neural processes ultimately enhancing reward salience of signals (including food) in the immediate environment.^35^ However, it should be noted that this hypothesis is still in the speculative stage of proposal and requires additional empirical evidence for corroboration.

The hypothesis that binge eating is partially driven by heightened reward salience, in combination with the finding that oxytocin increases vigilance to food stimuli, is difficult to consolidate with previous findings demonstrating that oxytocin decreases hedonic food consumption.^24^ That is, one might reasonably hypothesise that reduced hedonic food consumption would be associated with less attentional and motivational orientation to food stimuli. However, previous studies finding a suppression of hedonic eating reported this effect in male participant samples,^20^, ^24^, ^36^ although no effect of oxytocin on feeding was found in the current sample of female participants.^31^ An accumulating range of studies has found mixed effects of oxytocin on eating in women, both with and without eating disorders,^17^, ^27^ therefore suggesting that the inhibitory effect of oxytocin on hedonic eating and orienting to food stimuli may be sex‐specific in humans. However, further research is necessary to clarify whether oxytocin may influence reward salience attribution and actual food consumption via different mechanisms.

There is evidence to suggest that the potency of oxytocin on psychosocial functioning has an inverse quadratic function with drug dose.^37^ Given the relatively high dose of oxytocin administered in the present study (a divided dose of 64 IU), it may therefore be the case that oxytocin administration does have different effects on attentional bias in this population at lower doses. Previous evidence has indicated that the effects of oxytocin on resting neural activation vary over time.^38^ Therefore, it is possible that the functional effects of oxytocin on attentional biases to palatable food may also differ at other time points after administration given differing bioavailability of exogenous oxytocin to neural regions underpinning the control of attentional bias.

Additional limitations of the present study include the low sample size and our inability to measure the effects of oxytocin on early and late stages of attentional processing as a result of the nature of the dot probe task. It should be noted that a different pattern of effects may be found at different points within the time course of attentional processing given that different patterns of attentional vigilance vs avoidance have been observed at different stages of attentional processing in other eating disorders.^18^ Future studies of attention bias in BN and BED may be improved through the use of alternative measures of attentional bias, such as eye‐tracking tasks. Additionally, because oxytocin has been found to have sex‐specific effects in other studies of psychopathology,^39^ the findings of the present study should therefore not be generalised to men with BN and BED.

Finally, although we recruited women with BN and BED within our clinical sample in the present study, given that the primary trait of interest, recurrent loss‐of‐control binge eating behaviour, occurs in both disorders, it is worth noting that populations with BN vs BED differ in important ways. For example, people with BN are more likely to have a prior history of anorexia nervosa, ^40^ and people with BED are more likely to be overweight or obese. ^41^ Additionally, there is a greater degree of evidence to support elevated risk of obsessive‐compulsive behaviours and borderline personality disorder in BN, compared to BED. ^42^, ^43^ Therefore, these differences in aetiology and clinical profile, in combination with the divergence in the effects of oxytocin observed in the present study, suggest that it would be preferable to research populations with BN and BED separately in future.

In conclusion, to our knowledge, the present study is the first to investigate the influence of oxytocin on attentional bias to food images in women with BN and BED and healthy comparison women. A divided dose of 64 IU of intranasal oxytocin increased vigilance to palatable food images, although this effect is moderated by baseline attentional bias toward food images in the placebo condition. Further studies testing the effects of oxytocin on attentional biases to palatable food at different doses and time courses of administration in larger samples are warranted.

## CONFLICT OF INTEREST

The authors declare that they have no conflicts of interest.

## Supporting information

Supplementary Material

## Data Availability

The data that support the findings of the present study are available from the corresponding author upon reasonable request.
