# Supplementary material for: A pilot study investigating the influence of oxytocin on attentional bias to food images in women with bulimia nervosa or binge eating disorder
Source: J Neuroendocrinol. 2020 Mar 23;32(5):e12843. doi: 10.1111/jne.12843 (PMC8650572; doi:10.1111/jne.12843)
Supplement: Supplementary file 1 — Supplementary Material [file JNE-32-e12843-s001.docx]

**Supplementary Material**

**Appendix S1**

*Participant Inclusion and Exclusion Criteria*

Inclusion criteria for the study required participants to be female, aged between 18 and 40 years old, display English fluency, and to be right-handed (due to an MRI scan which was conducted within the battery of tasks). Exclusion criteria included pregnancy, severe comorbidity (e.g., substance abuse, drug addiction, psychosis, diabetes), history of drug dependence, history of a neurological condition (e.g., epilepsy), a significant visual impairment not corrected by eyewear, currently suffering from a cold or flu, currently smoking > 5 cigarettes per day (past 6 months), consuming > 21 units of alcohol per week, contraindication to MRI scans (due to an MRI conducted as part of the current battery of tasks), and current intake of medication that might potentially interact with oxytocin (e.g., Prostaglandins).

*Clinical Characteristics of the Participant Sample*

Participants with bulimia nervosa and binge eating disorder reported an average binge eating frequency of 14.14 episodes over the past 28 days (*SD* = 9.88). The women with bulimia nervosa endorsed an average frequency of self-induced vomiting equal to 10.40 occasions over the past 28 days (*SD* = 13.61), an average laxative abuse frequency of 5.13 occasions over the past 28 days (*SD* = 8.35), an average frequency of “hard exercise intended to control weight or shape” equal to 7.31 occasions over the past 28 days (*SD* = 8.57), and one participant reported using diuretic pills on 4 occasions over the past 28 days. Participants with bulimia nervosa or binge eating disorder reported having an eating disorder for an average of 10.30 years (*SD* = 5.87 years), with an average age of onset of 15.74 years (*SD* = 4.76 years).

**Supplementary Table 1**

*Results of the linear mixed effects analysis testing the moderating effect of hormonal state on the effects of oxytocin on attentional bias to food images*

| **Fixed Effects** | Estimate | *SE* | *df* | *t* | *p* |
| --- | --- | --- | --- | --- | --- |
| Intercept | 12.22 | 10.90 | 108.38 | 1.12 | .265 |
| Follicular Phase | -5.16 | 4.80 | 107.94 | -1.08 | .285 |
| Drug Condition | 3.12 | 13.92 | 135.90 | 0.22 | .823 |
| Follicular Phase*Drug Condition | 2.82 | 6.11 | 135.51 | 0.46 | .645 |
| **Random Effects** | Variance |  |  |  |  |
| Individual Participant | 156.30 |  |  |  |  |
| Residuals | 1273.20 |  |  |  |  |

**Supplementary Table 2**

*Results of the linear mixed effects analysis testing the moderating effect of visit number on the effects of oxytocin and eating disorder status on attentional bias to food images*

| **Fixed Effects** | Estimate | *SE* | *df* | *t* | *p* |
| --- | --- | --- | --- | --- | --- |
| Intercept | 4.54 | 15.20 | 153.65 | 0.30 | .766 |
| Visit Number | -4.37 | 9.52 | 165.74 | -0.46 | .647 |
| Drug Condition | 8.40 | 19.28 | 52.68 | 0.44 | .665 |
| Eating Disorder Status | 13.18 | 16.47 | 181.64 | 0.80 | .425 |
| Visit Number*Drug Condition | 0.95 | 12.45 | 46.09 | 0.08 | .940 |
| Visit Number*Eating Disorder Status | -5.03 | 10.23 | 142.30 | -0.49 | .624 |
| **Random Effects** | Variance |  |  |  |  |
| Individual Participant | 153.40 |  |  |  |  |
| Residuals | 1269.90 |  |  |  |  |

**Supplementary Table 3**

*Results of the linear mixed effects sensitivity analysis testing the main effects of eating disorder status, oxytocin, and food presentation (experiment time point) on attentional bias to food images among healthy control and BN participants*

|  |  | HC (*n* = 27)  Mean(SD) | BN (*n* = 25^†^)  Mean(SD) | Fixed Effects | |
| --- | --- | --- | --- | --- | --- |
| Before Taste Test | Oxytocin | 10.07(37.200) | 13.07(41.827) | ED Status: *Z* = 9.39, *SE* = 5.662, *df* = 45.64, *p* = .104  Drug Condition: *Z* = 8.11, *SE* = 4.662, *df* = 141.79, *p* = .084  Experiment Time Point: *Z* = -0.17, *SE* = 4.667, *df* = 143.35, *p* = .971 | |
|  | Placebo | -0.53(38.897) | 7.81(49.470) |  |  |
| After Taste Test | Oxytocin | 2.74(31.922) | 20.09(32.629) |  |  |
|  | Placebo | -0.26(26.770) | 6.93(35.809) |  |  |
|  |  |  |  | Random Effects | Variance |
|  |  |  |  | Individual Participant | 128.6 |
|  |  |  |  | Residuals | 1057.9 |

*Note.* BN = Bulimia nervosa; ED = Eating disorder; HC = Healthy control.

^†^ Data for five participants was simulated based on the imputed mean for each drug condition and experiment time point among the participant group with bulimia nervosa

**Supplementary Table 4**

*Results of the linear mixed effects analysis testing the effect of calories consumed in the taste status on attentional bias to food images*

| **Fixed Effects** | Estimate | *SE* | *df* | *t* | *p* |
| --- | --- | --- | --- | --- | --- |
| Intercept | 1.29 | 19.36 | 161.05 | 0.07 | .947 |
| Calorie Consumption | -0.01 | 0.04 | 159.49 | -0.17 | .862 |
| Drug Condition | 13.12 | 27.63 | 144.04 | 0.48 | .636 |
| Experiment Time Point | 3.84 | 12.22 | 142.58 | 0.32 | .754 |
| Calorie Consumption*Drug Condition | -0.01 | 0.06 | 144.67 | -0.09 | .929 |
| Calorie Consumption*Experiment Time Point | -0.01 | 0.03 | 141.50 | -0.26 | .798 |
| Drug Condition*Experiment Time Point | -3.70 | 17.66 | 142.79 | -0.21 | .834 |
| Calorie Consumption*Drug Condition*Experiment Time Point | 0.01 | 0.04 | 142.09 | 0.24 | .808 |
| **Random Effects** | Variance |  |  |  |  |
| Individual Participant | 143.00 |  |  |  |  |
| Residuals | 1283.00 |  |  |  |  |

*
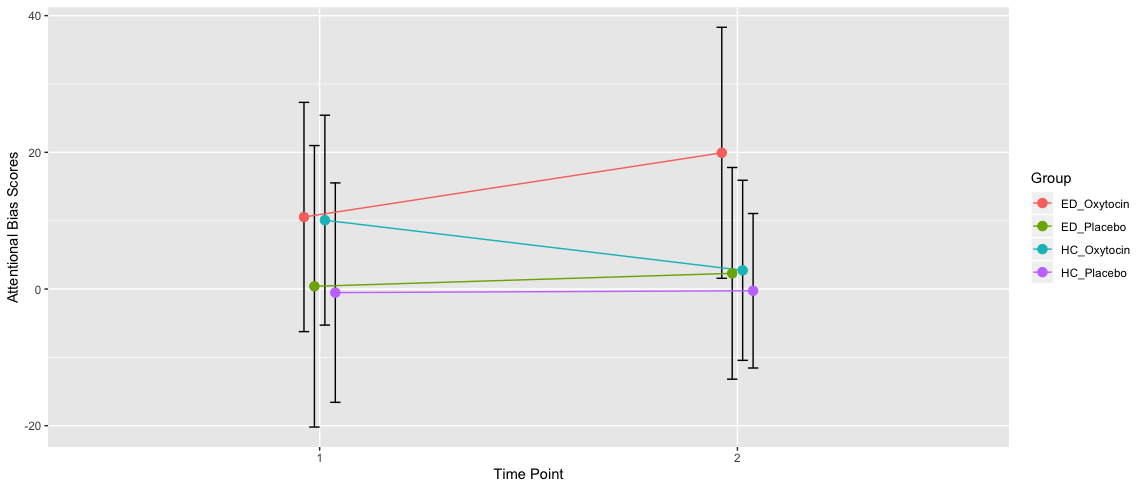
*

*Supplementary Figure 1.* The effect of oxytocin on attentional bias to food images. Time point 1 occurred after the full dose of oxytocin or placebo had been administered, but prior to the taste test. Time point 2 occurred after the taste test. Attentional bias scores were calculated for the dot probe task by subtracting each participant’s mean reaction time, in milliseconds, to probes that were preceded by a food image from those that were preceded by a neutral image. Error bars correspond to confidence intervals.


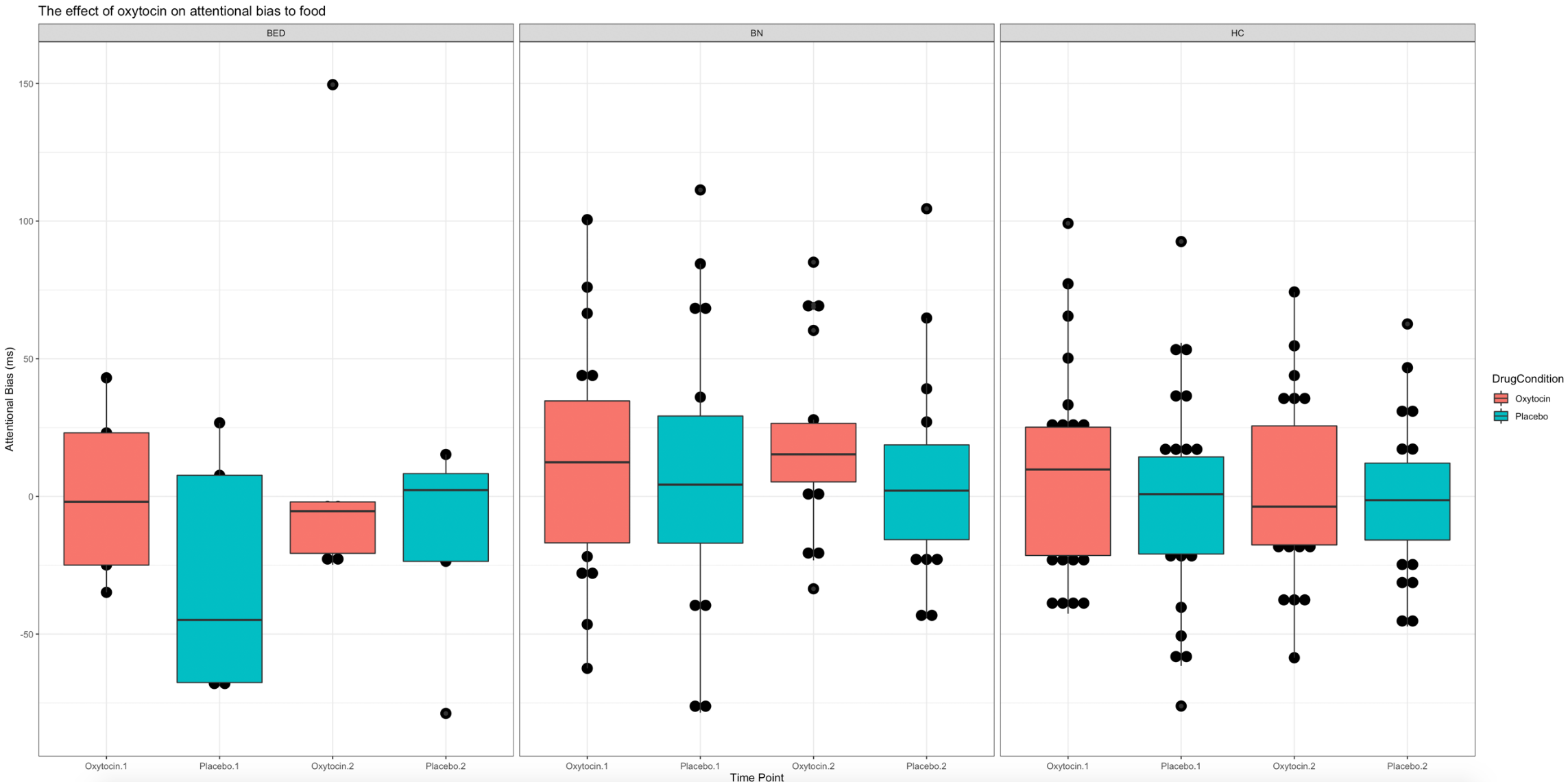


*Supplementary Figure 2.* A box and dotplot of the attentional bias data among each diagnostic group, separated by drug condition.


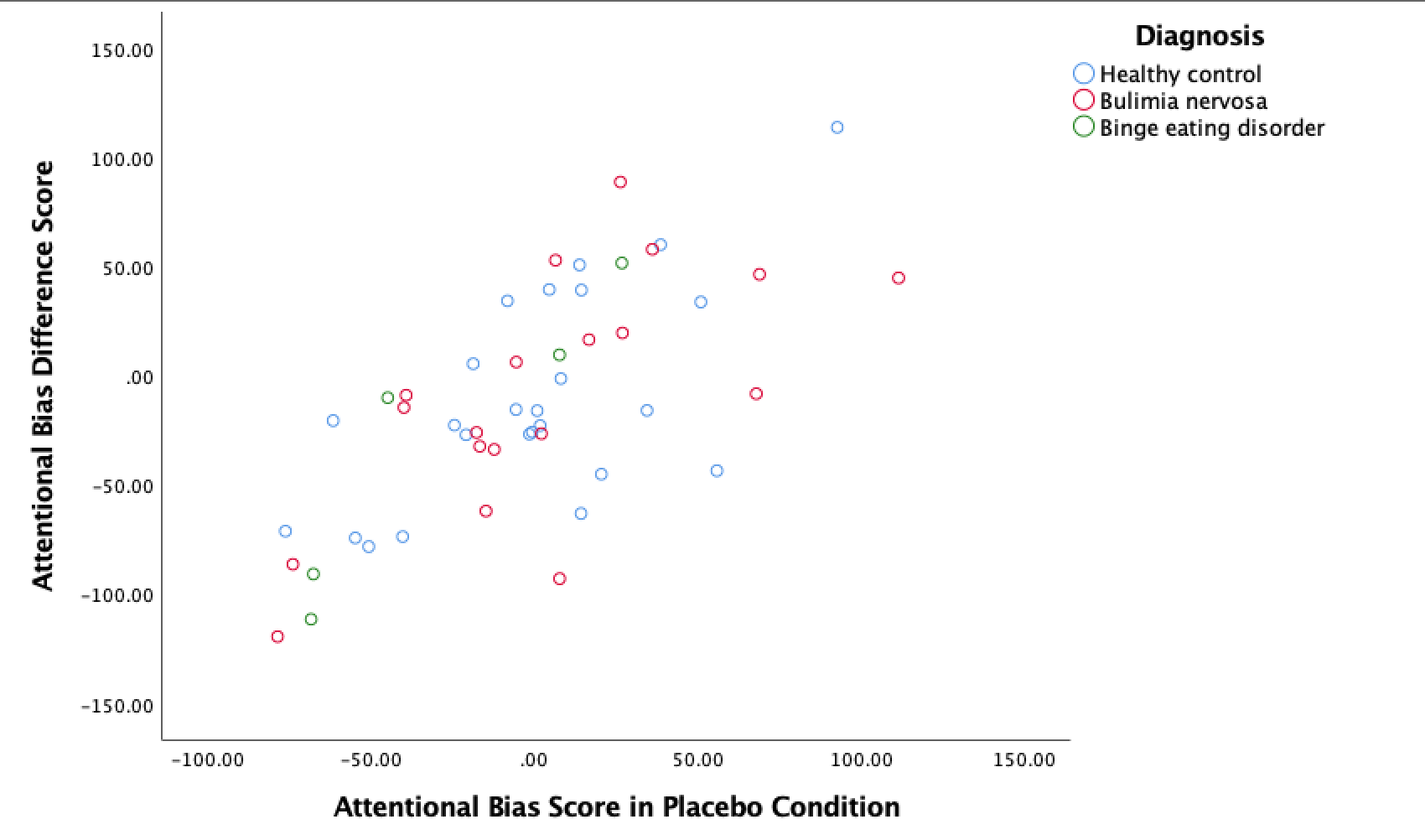


*Supplementary Figure 3.* Correlation between attentional bias score in the placebo condition in the placebo condition and the increase in vigilance caused by oxytocin at Time Point 1. Attentional bias scores were calculated for the dot probe task by subtracting each participant’s mean reaction time, in milliseconds, to probes that were preceded by a food image from those that were preceded by a neutral image.


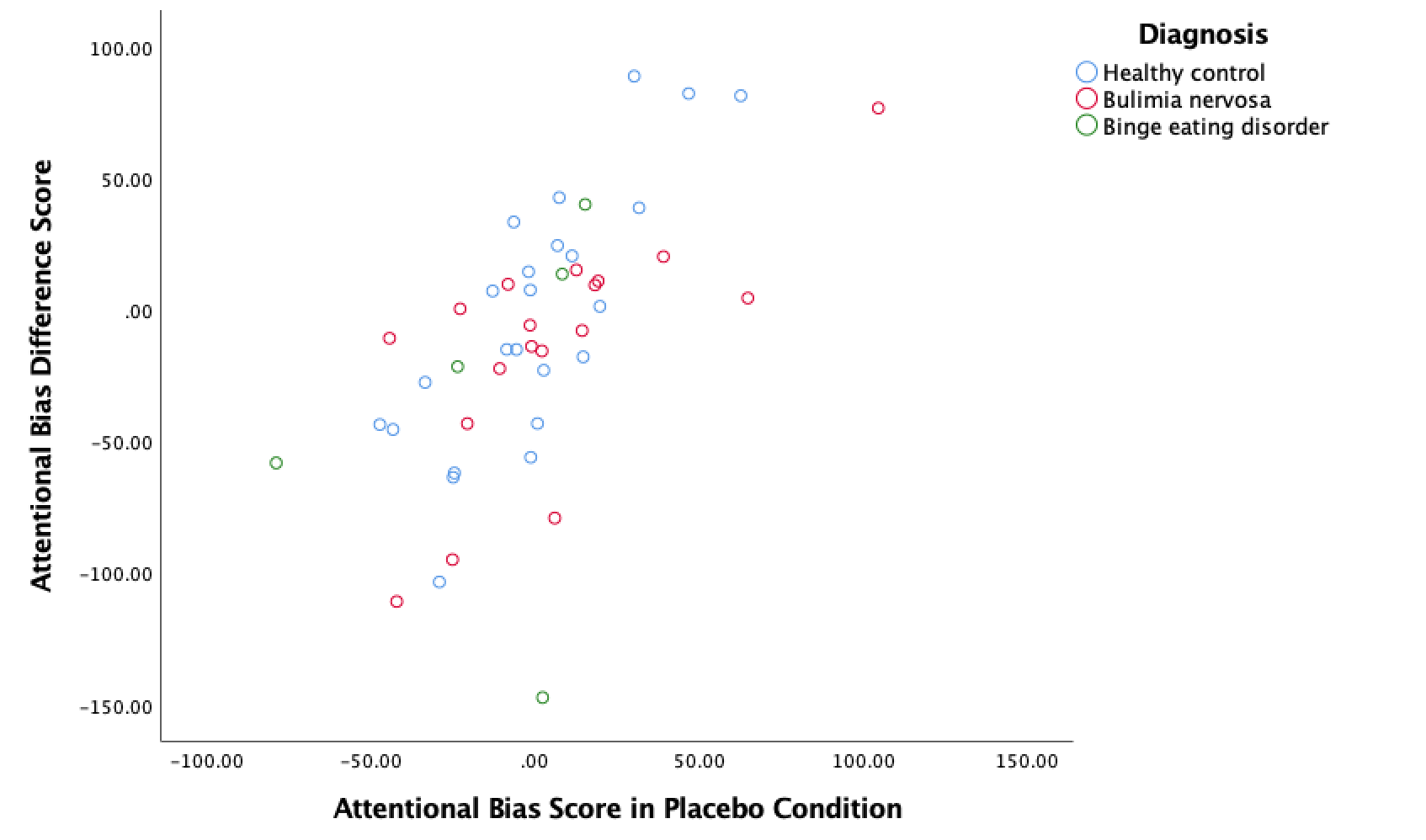


*Supplementary Figure 4.* Correlation between attentional bias score in the placebo condition in the placebo condition and the increase in vigilance caused by oxytocin at Time Point 2. Attentional bias scores were calculated for the dot probe task by subtracting each participant’s mean reaction time, in milliseconds, to probes that were preceded by a food image from those that were preceded by a neutral image.
